# Analysis of Web-Based IoT through Heterogeneous Networks: Swarm Computing over LoRaWAN

**DOI:** 10.3390/s22020664

**Published:** 2022-01-15

**Authors:** Samira Afzal, Laisa C. C. De Biase, Geovane Fedrecheski, William T. Pereira, Marcelo K. Zuffo

**Affiliations:** Laboratory for Integrated Systems, Department of Electronic Systems Engineering Polytechnic School, University of São Paulo (USP), São Paulo 05508-020, Brazil; laisa.costa@usp.br (L.C.C.D.B.); geovane@usp.br (G.F.); william.takeshi@usp.br (W.T.P.); mkzuffo@usp.br (M.K.Z.)

**Keywords:** swarm computing, LPWAN, LoRaWAN, interoperability

## Abstract

The Internet of Things (IoT) leverages added valued services by the wide spread of connected smart devices. The Swarm Computing paradigm considers a single abstraction layer that connects all kinds of devices globally, from sensors to super computers. In this context, the Low-Power Wide-Area Network (LPWAN) emerges, spreading out connection to the IoT end devices. With the upsides of long-range, low power and low cost, LPWAN presents major limitations regarding data transmission capacity, throughput, supported packet length and quantity per day limitation. This situation makes LPWAN systems with limited interoperability integrate with systems based on REpresentational State Transfer (REST). This work investigates how to connect web-based IoT applications with LPWANs. The analysis was carried out studying the number of packets generated for a use case of REST-based IoT over LPWAN, specifically the Swarm OS over LoRaWAN. The work also presents an analysis of the impact of using promising schemes for lower communication load. We evaluated Constrained Application Protocol (CoAP), Static Context Header Compression (SCHC) and Concise Binary Object Representation (CBOR) to make transmission over the restricted links of LPWANs possible. The attained results show the reduction of 98.18% packet sizes while using SCHC and CBOR compared to HTTP and JSON by sending fewer packets with smaller sizes.

## 1. Introduction

The Internet of Things (IoT) usually refers to interconnected heterogeneous objects (physical and virtual objects, smart objects, embedded computers, user interfaces, etc.) to collect and exchange data, establishing a smart environment [1]. IoT services are known as Smart Services, such as smart home environments, smart vehicles, smart cities, and smart transportation systems. However, it is not easy to have a comprehensive definition of IoT. Minerva et al. [2] define IoT in various contexts and concluded that the IoT is a heterogeneous ecosystem with major connectivity requirements for end devices. Therefore, in some IoT infrastructure examples, such as Swarm [3], no matter what the employed communication technologies are, there is a significant need for end devices to be accessible by any other end devices. The Swarm is a decentralized IoT framework of heterogeneous smart objects that share resources while providing QoS guarantees [4]. In particular, devices in Swarm offer their resources as services to explore the system synergy.

A class of network technologies that emerged for IoT communication is the Low-Power Wide-Area Network (LPWAN) [5]. LPWAN successfully presents long-range connectivity of low power end devices by offering adaptive transmission rates, transmission power, modulation, duty cycles, etc. LPWAN-based solutions considerably increase the range of common communication technologies based on the wireless sensor network (WSN) [6]. For example, LoRaWAN, which belongs to the LPWAN network category, has recorded 766 km (476 miles) [7]. Therefore, LPWAN proved to be a better fit for large WSN deployments than low-power short-range technologies or cellular alternatives [8,9]. While LPWAN shows benefits of high scalability, power efficiency, and reduced cost of end devices, it uses extremely constrained radio links with minimal packet sizes. Furthermore, currently, the available plans for LPWAN communications are limited in terms of the number of packets for downlink and uplink.

On the other hand, there is growing attention being paid to using web services and Representational State Transfer (REST) for IoT systems, since they can reduce applications’ complexity and improve software reusability [10]. REST [11,12] is a software architectural style that defines constraints for distributed hypermedia systems, such as the web, to drive the design decisions towards enhancing the quality attributes. REST has been a de facto architecture style for web-based systems. The most commonly used communication protocol for the RESTful application is the Hypertext Transfer Protocol (HTTP) [13]. In the REST architectural style, each component is assumed as a resource where they can be accessed by a common interface utilizing general CRUD commands. However, HTTP may cause fragmentation in constrained networks because of its size, which reduces its throughput.

Another typical scheme for RESTful application is Constrained Application Protocol (CoAP) [14]. CoAP is an application-layer communication protocol recommended for constrained scenarios since it is lightweight compared to HTTP. Although CoAP packet sizes could be reduced by over 90% at best compared to HTTP, the CoAP headers could yet be large for the LPWAN links since it is over the IP protocol. For this reason, another effort of the IETF’s LPWAN workgroup was to encapsulate CoAP/UDP/IP by applying the Static Context Header Compression (SCHC) scheme [15], which offers header compression and fragmentation. Additionally, Concise Binary Object Representation (CBOR) [16] is a data format recommended for constrained network scenarios, designed for small code size and small packet size. CBOR modifies the JavaScript Object Notation (JSON) [17] data model by allowing for binary data. Using such schemes could decrease the packet sizes and reduce or eliminate the need for fragmentation.

This work investigates the compatibility of REST-based IoT with LPWANs. We construct and present a network architecture for interconnecting the constrained LPWAN networks with web-based IoT infrastructure networks. The analysis was carried out studying the size and number of packets generated for a use case of REST-based IoT over LPWAN, specifically the Swarm over LoRaWAN. We analyze the communication load by studying different schemes to achieve interoperability. The communication load impact of different schemes of CoAP, SCHC and CBOR for Swarm and LoRaWAN network use cases are evaluated through different approaches:

- Using CoAP protocol for header and JSON serialization format for payload;
- Using CoAP protocol for header and CBOR serialization format for payload;
- Using SCHC compression technique for header and CBOR serialization format for payload.

We also analyze the impact of the CBOR Object Signing and Encryption (COSE) security service on the communication load for Swarm communication with limited data transmission LoRaWAN:

- Using CoAP protocol for header and CBOR serialization format for payload with COSE security service;
- Using SCHC compression technique for header and CBOR serialization format for payload with COSE security service.

Analysis results show significant communication load reduction of 98.18% in terms of packet sizes while using SCHC and CBOR compared to HTTP and JSON by sending fewer packets with smaller sizes through the constrained data transmission LoRaWAN. Even the approach using SCHC and CBOR with the applied secure COSE scheme can decrease by up to 68.31% in terms of packet sizes compared with using HTTP and JSON schemes.

The rest of the work is structured as follows: Section 2 contains a review of the different approaches used to integrate web-based IoT infrastructure with constrained networks. Section 3 introduces the background on LoRaWAN technology, CoAP and SCHC schemes, JSON and CBOR data formats. Section 4 presents the Swarm use case and contains a description of the proposed network architecture used to evaluate the Swarm communication load with limited data transmission LoRaWAN considering different schemes. Finally, Section 5 concludes with the analyzed results and identifies future lines of research.

## 2. Related Work

The transmission of IP packets over LPWAN networks has received the interest of researchers in recent years—Table 1. Works [18,19] have enhanced IPv6 over Low power WPAN (6LoWPAN) to address the integration of higher-layer protocols within LPWAN architectures. 6LoWPAN is a network layer adaptation protocol that adapts IPv6 to IEEE 802.15.4 networks.

Thielemans et al. (2017) [18] proposed an approach by encapsulating IPv6 packets over LoRa. In this work, the Contiki operating system is extended to support of LoRa replacing the LoRaWAN MAC protocol with an IPv6-based networking stack. This networking stack allows LoRa-based point-to-point connectivity and direct IPv6 communication without the demand of application gateways.

The proposed solution in [19], named 6LoRaWAN, presents two different mechanisms to fit the IPv6 header within the LoRaWAN Maximum Transmission Unit (MTU). One mechanism is a static concept based on a previously stored transformation agreement between the end device and the gateway. The other one is a dynamic concept implying a previous negotiation. However, since these above-mentioned works are based on the IPv6 over 6LoWPAN adaptation approach implies protocol overhead, higher data rates and shorter ranges than LPWAN networks [23].

In 2016, the IETF formed the IPv6 over the LPWAN working group considering UDP and CoAP protocols over LPWAN networks. The documents [15,24] propose the Static Context Header Compression (SCHC) to allow the adaptation of IPv6/UDP/CoAP headers for transmission over the restricted links of LPWANs. A detailed exploration of these mechanisms will be presented in the following sections.

In [20,21,22] addressed the integration of higher-layer protocols within LPWAN architectures and explored the performance of the SCHC compression and fragmentation scheme. Sanchez-Gomez et al. (2020) [20] implemented a real testbed for a LoRaWAN-to-IPv6 architecture together with a publish/subscribe broker for CoAP. Different SCHC and LoRaWAN configurations (e.g., data rates) are analyzed in this work. The attained results show the advantages of SCHC on the performance with the great benefit of reducing the Time on Air (ToA) and also important improvement in the reliability of the LPWAN links when the data rate is lower, in other words when compression is higher. However, it is proven that fragmentation can impose a lack of efficiency in terms of data and energy.

Lara et al. (2019) [21] conclude that there is a non-linear relationship between ToA and the percentage of compression of each rule in SCHC. Abdelfadeel et al. (2017) [22] evaluate SCHC against 6LoWPAN compression, assessing the improvement in compression rate over LoRaWAN. This work enhanced SCHC with layered context called Layered SCHC (LSCHC). LSCHC reduces memory consumption and processing complexity compared with the SCHC, and adds more flexibility when compressing packets. Sanchez-Iborra et al. (2018) [23] complemented this with a security extension based on the Ephemeral Diffie–Hellman Over COSE (EDHOC) protocol. EDHOC messages are protected by COSE and are encoded following CBOR. EDHOC makes the configuration of nodes easier to avoid the manual distribution of keys and even improves the performance when key updates are necessary using the end device, compared with the current security features of LoRaWAN.

The key drawback of these works is that the fragmentation capacities of SCHC are not utilized and not evaluated. In contrast with the related works, we focus on investigating the communication load studying the header and payload size of packets and the number of packets generated for a use case of REST-based IoT over LPWAN, specifically the Swarm over LoRaWAN. We evaluate comprehensively different schemes of CoAP and SCHC, CBOR and JSON serialization formats and the COSE security service.

## 3. Background

### 3.1. Restrictions in Low-Power Wide-Area Network-Based Technologies

LPWAN networks have major limitations regarding data-transmission capacity and number of packets for transmission. LPWAN has a constrained Layer 2 (L2) payload size, which is from tens to hundreds of bytes [20]. This is the main reason that LPWAN systems usually do not offer direct connectivity of end devices to the web-based IoT infrastructure. In particular, LPWAN has a tiny L2 MTU compared to the IP MTU, which is 1280 bytes. For example, the maximum payload size for a packet in LoRaWAN technology is 242 bytes [25], which could be achieved only under specific conditions such as the EU 868 ISM global region data rate 5 or AU 915 ISM data rate 4 and 5 and without repeaters. In other conditions and data rates, it is even more constrained and the maximum payload size for a packet was only 51 bytes.

LoRaWAN is a constrained LPWAN network with attractive specific features and market perspectives in the context of IoT services. LoRaWAN [26] refers to the long-range wireless network technology supported by well-known corporations such as Cisco and IBM.

There are two layers involved in this technology: (i) a physical layer LoRa radio and (ii) a MAC layer protocol (LoRaWAN). The physical layer uses LoRa radio technology and employs the Chirp Spread Spectrum (CSS). LoRa is a proprietary radio technology developed by Semtech for long-range, low-power, and low-throughput wireless communications. The MAC layer, called LoRaWAN, provides a medium access control scheme and empowers IoT devices to communicate with the gateway utilizing the LoRa modulation. LoRaWAN is an open standard developed by LoRa Alliance [27]. Each LoRaWAN message consists of a mandatory 13-byte header and different maximum message payloads depending on the ISM and data transfer configuration. Therefore, fragmentation in LoRaWAN should be avoided in order to reduce the intrinsic overhead associated with each packet. Finally, the maximum payload size for a packet in LoRaWAN technology is 242 bytes the under specific conditions previously mentioned.

**Figure 1:**
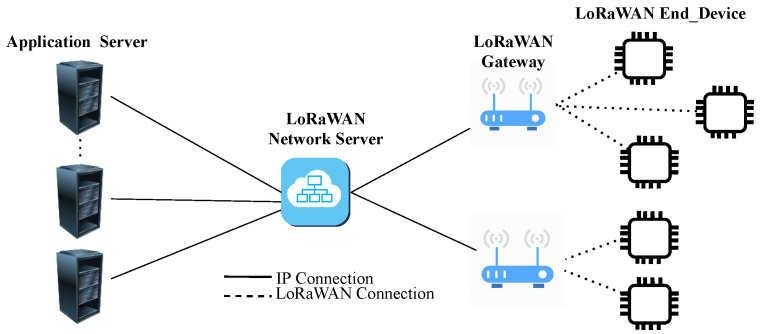
Overview of the LoRaWAN network architecture.

A typical LoRaWAN network architecture consists of end devices, gateways, network servers, and application servers. As exemplified in Figure 1, end devices are connected via the LoRa radio to one or more LoRaWAN gateways. The gateways are bridges between end devices and network servers to forward packets from the end devices to the network server or vice versa. The network server is responsible for forwarding uplink packets to the appropriate application servers and also forwarding the downlink packets coming from any application server to the associated end devices. The network server also checks device addresses, manages acknowledgements, controls frame counter and frame authentication.

### 3.2. CoAP

Constrained Application Protocol (CoAP) [14] is a specialized web transfer protocol typically on top of UDP/IP to simplify the network stack, enabling its implementation on constrained communication networks such as LPWAN. This RESTful application protocol provides the same basic set of HTTP services, such as using the same address space, caching scheme and methods. CoAP is similar but less complex than HTTP. Additionally, CoAP specification allows interactions based on simple request/response transactions. It uses the REST or HTTP methods, such as GET, PUT, POST, and DELETE. CoAP works well with IPv6 and with most of today’s IPv4 networks. Readers can refer to [28] for more information regarding CoAP protocol for tiny internet devices.

### 3.3. SCHC

The IETF’s LPWAN working group has proposed a Static Context Header Compression (SCHC) scheme that allows compression and fragmentation of CoAP/UDP/IPv6 packets intending to make them suitable for transmission over LPWANs. In particular, the IPv6 protocol used in web-based IoT infrastructure has an MTU of 1280 bytes. In this way, a single transmission of one IPv6 packet would need several fragments simply for sending the IPv6 header. To solve the aforementioned issues, the SCHC scheme is developed as an adaptation layer to compress the header and fragment the packets if needed. It is worth mentioning that compression and decompression require processing power, increasing the communication delay [20].

### 3.4. Data Formats JSON and CBOR

Two common data formats in IoT are JSON and CBOR. Data format refers to language-independent data structure representation that offers a common syntax and scheme for different data types and structures [29]. JavaScript Object Notation (JSON) [17] is a popular text-based format easy for humans to read and write. JSON is widely used for data transmission between web services. The format is natively supported by JavaScript to serialize JavaScript objects [30].

Another scheme is Concise Binary Object Representation (CBOR) [16]. CBOR is based on a JSON data model by allowing for binary data serialization. CBOR is a standard effort specifically designed for the small, constrained devices of IoT technologies with major data-transmission limitations. Therefore, it offers small message transport and implementation size without the need for version negotiation [31]. This leads to speeding up of the processing and transfer at the cost of human readability.

## 4. Use Case

The use cases used for this architecture are LoRaWAN as a constrained LPWAN network and Swarm [32] as an example of web-based IoT infrastructure.

**Figure 2:**
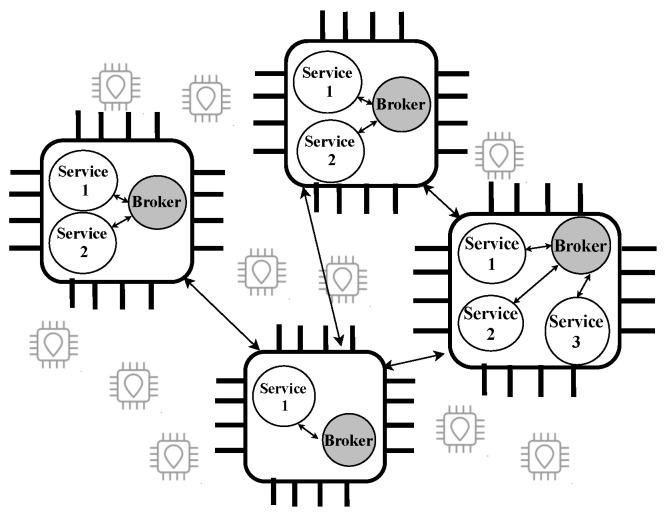
An example of decentralized structure of the Swarm network.

Swarm is a self-adapted IoT network for distributed intelligent objects, from sensors to super computers, heterogeneity, variable computing power, and energy capabilities. Swarm manages interaction among the participants to share resources among them. In Swarm, each device contains a key component, Broker (platform service), and one or more application services to facilitate communication among application services. Application services are all other services that cooperate in the Swarm. Figure 2 illustrates a general structure of the Swarm consisting of IoT devices. In this example, application services are briefly named services, and these services have interaction with the Broker and among each other. The Swarm network behaves with an organized behavior in an emergent collective intelligence. We refer the reader to Biase et al. (2018) [3] for more technical details on the platform service.

Swarm offers IoT services in the form of service consumers and service providers, which are types of software agents that interact among themselves in the microservices ecosystem [3]. In particular, the service consumer running on the consumer device requests a task from the service provider running on the provider device. While a service consumer can consume more than one service, each service provider can provide only one service. The Swarm Broker is associated with these services and facilitates transactions.

Generally, the transactions in the Swarm follow nine steps which are briefly explained below.

(1) Registration: Each Service Provider registers with its corresponding local Broker defining the service functionality and usage conditions (QoS and Service price). This way, the service would be available for sharing.
(2) Service Request: The Service Consumer requests a service to its associated Broker, defining its required QoS and the maximum Service Price.
(3) Discovery: The Service Consumer’s Broker searches for finding Service Provider candidates that match the requested requirements.
(4) Optimum Service Provider Selection: The Service Consumer’s Broker chooses the best suitable offered service provider that provides the required service functionality (QoS and Service price).
(5) Negotiation: The next step is creating a transaction representing a signed money transfer and Service Level Agreements (SLA), including information such as the Service Consumer, the Service Provider, and conditions for service execution. The SLA should be agreed upon with both Service Consumer and Service Provider’s Brokers.
(6) Payment: The Service Consumer’s Broker will transfer the credits in order to pay for the transaction.
(7) Service Level Agreement: After the payment is approved, access permission to resources will be created, as per the SLA.
(8) Feedback and reputation point attribution: The Service Consumer’s Broker and the Service Provider’s Broker give reputation points to each other based on characteristics of the transaction flow. The Service Consumer also evaluates the Quality of the provider Service.
(9) Use: The service customer communicates directly with the service provider.

### 4.1. Proposed Network Architecture

**Figure 3:**
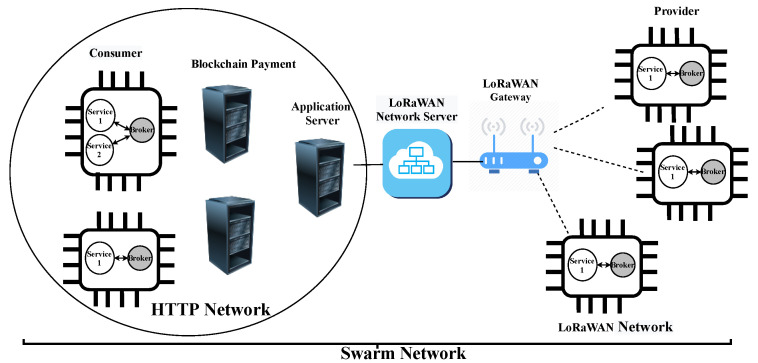
Overview of the proposed network architecture.

Figure 3 depicts the proposed architecture for interconnecting LoRaWAN and Swarm networks. In this proposed architecture, the LoRaWAN end devices run one Broker beside the application services. This installed Broker makes LoRaWAN end devices a part of the Swarm network. Furthermore, the application server bridges the HTTP network with the LoRaWAN network. Noting that the application server acts as a proxy server to perform cross-protocol conversion in the testing scenarios of Section 4.2, Section 4.3, Section 4.4 and Section 4.5. In this example, as shown in the Figure 3, LoRaWAN end devices act as providers and the Swarm devices in the HTTP network act as consumers. This proposed network architecture could be used for future integration of web-based IoT infrastructure with other constrained LPWAN architectures.

**Figure 4:**
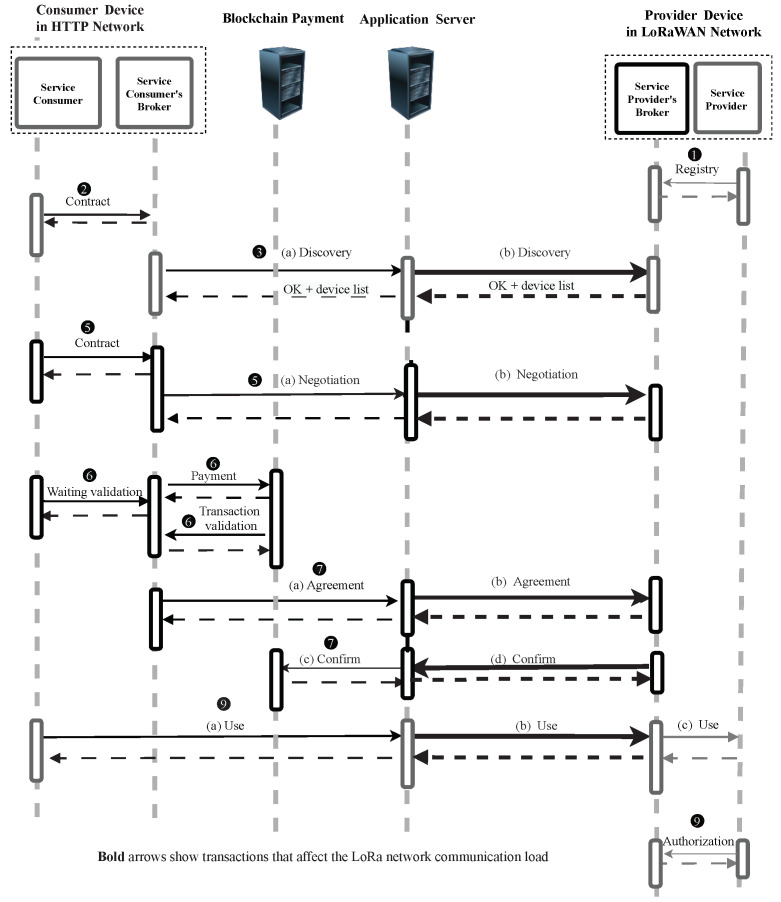
Sequence diagram of communication between services and networks.

Since Swarm is a RESTful based network using HTTP, TCP, and IPv4 in the network could produce large sizes. However, LoRaWAN is a constrained network which can only support only small-sized packets, which, as we previously mentioned, is in the limitation of the number of packets [25]. Therefore, here, we analyze the communication load over the LoRaWAN network when running the Swarm. Figure 4 presents the transactions that show communication between services and network communication. That is the reason why the steps “(4) Select optimum service provider” and “(8) Feedback and reputation points attribution” are not shown in this Figure. As mentioned above, the device connected to the LoRaWAN is a provider and the device in the HTTP network is a consumer. Bold arrows in this figure show the transactions that affect the LoRaWAN communication network which are steps “(3b) Discovery”, “(5b) Negotiation”, “(7b) Agreement (SLA establishment)”, “(7d) Confirm” and “(9b) Use”. Since these steps were proposed and implemented in a previous work [3], we re-ran them and used Wireshark (https://www.wireshark.org/, accessed on 21 December 2021) to measure the amount of bytes used by each protocol layer (i.e., IP, TCP, and HTTP).

Table 2 illustrates the communication load over the LoRaWAN side in our proposed architecture (transactions that are shown in bold arrows in Figure 4). In this work, all the analyses are undertaken under specific LoRaWAN conditions supporting a maximum of 242 bytes payload for each packet as previously explained in Section 3. Each LoRaWAN packet also has a mandatory 13-byte header, which is considered out of the scope of our analysis, since we are focusing on reducing the overhead of RESTful messaging in LPWAN environments. Swarm is a RESTful-based network that one of its implementation relies on HTTP, wraps the HTTP header, TCP header, IP header and payload. These related RESTful headers would consist in the LoRaWAN payload and, therefore, when this part is more than maximum limited payload, it needs to be fragmented. This table presents header sizes and payloads of packets for each transaction as well as total header and total packet sizes. One can see that the HTTP header forms a big part of the header, while TCP and IP headers have static sizes of, respectively, 32 and 20 bytes (we considered IPv4 for the network protocol communication). In our case, the HTTP header has significant size with different values for each transaction. This is because different parameters are set over different HTTP messages. Another note is that, as expected, we can see that packets have significant big sizes in total. For example, in the “(3) discovery”, the request packet has 474 bytes and the response packet has 1131 bytes. These sizes are much more than the LoRaWAN restriction packet size with the maximum possibility of only 242 bytes. This is the same for many packets of the rest of the transactions shown in the table. Furthermore, the table shows that the packets are big because they have both headers and payloads of significant size. For example, the request packet of the “(3) discovery” step has a total header of 283 bytes and a payload of 191 bytes. Thus, the header, alone, exceeds the limited data transmission of LoRaWAN. This is the same for many packets of the rest of the transactions that are shown in the table.

Most of the packet sizes expressively exceed the LoRaWAN restriction of 242 bytes. Therefore, the packets should be fragmented to a maximum of 242 bytes each. The table indicates high LoRaWAN fragments for some of the transactions. For example, the negotiation (5-b) request and response have five LoRaWAN fragments and confirm that the (7-d) response has six LoRaWAN fragments to send through the LoRa link.

It is also worth noting that the USE Req. (9-b) has 0 bytes of payload but it is fragmented into 2 pieces since the header is 261 bytes over 242 bytes.

Next, we present some alternatives for running the Swarm over LoRaWAN and evaluate their respective communication loads. In the first approach, we optimize header size by replacing HTTP/TCP/IP with the CoAP/UDP/IP header. Then, we optimize payload size by using format serialization of the CBOR scheme while using CoAP for the header. Next, we explore and evaluate SCHC header compression as long as the CBOR payload is used. Finally, we analyze the COSE communication load. In order to measure the different message sizes when changing to CoAP (https://github.com/chrysn/aiocoap, accessed on 21 December 2021), CBOR (https://github.com/agronholm/cbor2, accessed on 21 December 2021), COSE (https://github.com/TimothyClaeys/pycose, accessed on 21 December 2021), and SCHC (https://github.com/ltn22/SCHC, accessed on on 21 December 2021), we leveraged existing open-source libraries and analyzed the resulting sizes and the respective number of fragments.

### 4.2. Load Evaluation of Swarm Communication with Limited Data Transmission LoRaWAN Using CoAP

Here, we first explain why we selected CoAP as a scheme to evaluate with a constrained environment, then we will explain the proposed network architecture fit with this integration. Finally, we analyze the communication load over LoRaWAN using the CoAP protocol to see if it fits the restricted environment.

HTTP is a powerful and widely used protocol. However, what we really need in constrained environments is REST and not necessarily all HTTP’s features [28]. Therefore, this section evaluates CoAP for data transmission in the constrained environment, since it is suggested by the IETF Constrained RESTful Environments (CoRE) working group for constrained devices and networks. CoAP, as explained previously in Section 3, has the REST architecture, making it easy to implement and integrate with REST infrastructure networks. For example, both CoAP and HTTP use similar address space and caching techniques.

Furthermore, HTTP is designed to interoperate through proxies and it makes it easy to map the CoAP header to the HTTP header and vice versa. For this purpose, we use the application server to act as a proxy in the proposed network architecture illustrated in Figure 3. Proxy is an intermediary entity that performs cross-protocol conversion [10,33]. A proxy server can have a key role in consolidating common communication between different protocols, especially for interoperability in IoT devices.

Table 3 illustrates the Swarm communication load over LoRaWAN in our proposed architecture using the CoAP/UDP/IP scheme instead of HTTP/TCP/IP, previously presented in Table 2. The CoAP protocol wraps the packets with the CoAP header, UDP header, IP header, and payload. In this scenario, the CoAP header is assumed to be 15 bytes since it is typically between 10 and 20 bytes [28]. Therefore, the CoAP header is up to 95.5% smaller than the HTTP header. UDP is a lightweight protocol of 8 bytes only vs. 32 in TCP, which corresponds to a 75% reduction. Similar to the Table 2, the IP header has 20 bytes. Since all the headers in the CoAP/UDP/IP scheme have static values, the total header size in all the transactions of this scheme has the same size of 43 bytes. This value is significantly comparable with the HTTP/TCP/IP scheme’s total header size and reduces the header size at least 78.61%, and up to 88.83% in our scenario. For instance, the total header of CoAP/UDP/IP in the discovery (3-b) Req. step is reduced from 283 bytes to only 43 bytes, which corresponds to an 84.81% reduction. In turn, the reduced header size can make the total packet size up to 88.31% smaller. For instance, the total packet size of CoAP/UDP/IP in the discovery (3-b) Req. step is reduced from 474 bytes to 234 bytes, which corresponds to a 58.44% reduction. However, the total size of packets in some transactions is still much more than the LoRaWAN restriction packet size, which in the maximum case is only 242 bytes. Therefore, the packets should be fragmented into small packets of a maximum of 242 bytes. The table indicates a high number of LoRaWAN fragments for some of the transactions. For example, the negotiation (5-b) request and response and confirm (7-d) response require fragmentation into five packets to send through the LoRa link. Therefore, it is important to reduce not only the header size but also the payload of the packet to make it smaller and, consequently, produce a smaller number of LoRaWAN fragmentations. Next, while we keep the CoAP header, we will use the CBOR scheme for the packet payload and evaluate the impact of this scheme in the Swarm communication with LoRaWAN technology.

### 4.3. Load Evaluation of Swarm Communication with Limited Data Transmission LoRaWAN Using the CoAP Protocol and CBOR Technique

CoAP can carry different types of payloads. This section keeps the CoAP/UDP/IP header carrying the CBOR payload since it offers small message size transmission. Additionally, it is the recommended data format for the CoAP Internet of Things protocol suite [14] and has been adopted by several of the IETF working groups. The main features of CBOR are previously explained in Section 3. Here, we evaluate the impact of this scheme in the Swarm communication with LoRaWAN technology to see if it fits the restricted environment.

Table 4 illustrates the Swarm communication load over LoRaWAN in our proposed architecture using CBOR payload instead of JSON. It is easy to observe from the results that, in this scenario, a JSON payload compressed up to 50% showing the effectiveness of this method. Additionally, compared to HTTP/TCP/IP, shown in Table 2, the packet sizes are reduced up to 88.57%.

Next, we will use the SCHC compression technique for compressing the header size while we keep the CBOR payload to evaluate the impact of this scheme in the Swarm communication with LoRaWAN technology.

### 4.4. Load Evaluation of Swarm Communication with Limited Data Transmission LoRaWAN Using SCHC and CBOR

In this section, we replace the SCHC with CoAP/UDP/IP header explained in Section 4.3. It should be noted that SCHC reliability modes such as ACK-on-Error and ACK-always are not considered in our Analysis.

For compression of the CoAP/UDP/IP header, we apply the SCHC rule for all the header fields. One challenge here is that SCHC was designed for IPv6 and the current Swarm implementation uses IPv4. In this case, since the SCHC specification states that “in most cases a small Rule identifier is enough to represent the full IPv6/UDP headers”, we decided to test SCHC with IPv6. Regarding the CoAP header, SCHC can reduce it to 4 bytes [24]. SCHC Compression works as follows. First, the compressors perform the compression in the IPv4/UDP level, and therefore, the 20 bytes of the IP header and the 8 bytes of the UDP header are compressed by the Rule ID (1 byte). In the next level, the CoAP is compressed resulting in 4 bytes. These bytes correspond to the Message ID and Token field. Both ends of the communication can compress and decompress. In our example network architecture, presented in Figure 3, the application server and provider devices are both ends of the communication and they can communicate bidirectionally. Therefore, both can apply SCHC compression and decompression. In addition, the application server acts as a proxy to map the HTTP header to CoAP and vice versa. For example, the provider end device compresses the CoAP/UDP/IP packet to an SCHC packet and transmits it through the LoRaWAN network; then, the application server decompresses it to the original CoAP/UDP/IP packet. In case the destination address (the consumer device) is in an HTTP network, then the application server maps the CoAP/UDP/IP header to the HTTP.

If the SCHC packet size is larger than the LoRaWAN MTU after the SCHC compression is applied to the CoAP/UDP/IP packet by the end device, then the fragmentation process is applied to the packet. Finally, the SCHC fragments are sequentially sent as regular LoRaWAN messages through the radio link. This way, a Fragment Compression Number (FCN) (1 byte) will be added to the header. Thereafter, the other end of the communication must apply the correspondent reassembly and decompression context to obtain the original CoAP/UDP/IP packet. Since the application server acts as a proxy server, it can map the CoAP/UDP/IP to the HTTP/TCP/IP if needed. Moreover, the payload is compressed to the CBOR as we previously showed the effectiveness of this scheme in the Section 4.3. We remind the reader that CBOR compresses the payload up to 50% in our scenario.

Table 5 illustrates the communication network load over LoRaWAN in our proposed architecture (transactions that are shown in bold arrows in Figure 3). While the CBOR technique is used for payload compression, the SCHC compression technique is used to compress the header size. The table shows the packets’ parts which are SCHC header and CBOR payload. One can see that SCHC header has only 5 or 6 bytes for the reason already explained, and this is actually the total header size. Comparing with header sizes provided in Table 2 and Table 4, the SCHC header is up to 98.72% and 88.37% smaller than, respectively, the total HTTP/TCP/IP and CoAP/UDP/IP header sizes. In turn, the table also shows the compressed payload sizes by CBOR and the total packet sizes. It is clearly shown that the total sizes of packets in some transactions are still much more than the LoRaWAN restriction packet size which, as we mentioned before, in the maximum case is only 242 bytes. Therefore, the packets are fragmented considering the MTU and the number of fragments presented in the table.

A comparison between the results presented for the scenario of using the CoAP/UDP/IP header and CBOR payload in Table 4 with the results presented for the scenario of using the SCHC header and CBOR payload in Table 5 shows that, while the numbers of LoRaWAN fragments are not different, in the scenario of SCHC and CBOR, the length of each packet is smaller. This could affect delay and interference in the network.

The attained results show the advantages of SCHC on the performance.

### 4.5. Analysis of the COSE Communication Load for SwarmCommunication with Limited Data Transmission LoRaWAN

CBOR Object Signing and Encryption (COSE) is defined to create security services for the CBOR data format. COSE describes how to create and process encryption, signatures and message authentication codes using CBOR for serialization [34]. COSE is applied at the application layer of the network, can be maintained end-to-end and set on a per-message basis. While different algorithms can be used in COSE, we selected a specific set of algorithms according to our needs. To derive a session key, we employ ECDH-SS-HKDF-256, which uses an elliptic Diffie–Hellman curve with two static keys, along with a key derivation function based on SHA-256. The use of static keys implies that the sender does not need to transmit the public key used for key agreement, thus reducing message size. To encrypt based on the derived session key, we used the COSE algorithm AES-CCM-16-64-128, the Advanced Encryption Standard in CCM mode with a 64-bit tag and a 13-byte nonce. Furthermore, to provide non-reputability, we also employ a message signature using the algorithm EDDSA. It consists in the Edwards Curve Digital Signature Algorithm, a variant of Schnorr’s signature system with (possibly twisted) Edwards curves.

Using COSE would increase the size of packets in terms of the cost of secure communication. Please note that the combined COSE overhead for the algorithms mentioned above is 115 bytes.

The communication load for Swarm with LoRaWAN in our proposed architecture using the CoAP protocol and CBOR technique while secured with the COSE security service (transactions that are shown in bold arrows in Figure 3) is shown in Table 6. The total header is 158 bytes, which is made up of 115 bytes of COSE, 15 bytes of CoAP, 8 bytes of UDP and in turn 20 bytes of IP header. One can see that, compared to the HTTP/TCP/IP without COSE, all the packet sizes are reduced by up to 58.70%. Another result is that, compared to the scheme without COSE security service, Table 4, the extra COSE header affects on the number of LoRaWAN fragments. Therefore, there are 37.5% and 23.07% more uplink and downlink fragments, respectively.

Similarly, the communication load for Swarm with LoRaWAN in our proposed architecture considering the SCHC and CBOR techniques while secured with COSE is shown in Table 7. The total header is 121 bytes which is made up of 115 bytes of COSE, and 6 bytes of SCHC header. One can see that, compared to the HTTP/TCP/IP without COSE, all the packet sizes decreased by up to 68.31%. Compared to Table 5, the extra COSE header increased the number of LoRaWAN fragments. Therefore, there are 12.5% and 15.38% more uplink and downlink fragments, respectively.

## 5. Conclusions and Future Work

In this work, we analyzed the challenge of connecting LPWAN technologies to the web-based IoT infrastructure. First, we presented a network architecture for interconnecting the LoRaWAN and constrained the LPWAN network with Swarm in Section 4. In the proposed architecture, a broker service is installed on each LoRaWAN end device and an application server bridges the HTTP network with the LoRaWAN network.

Then, considering the limitations of LPWANs, we evaluated different schemes of CoAP, SCHC, and CBOR by defining different approaches in terms of network communication efficiency. In particular, we focused on reducing communication load in order to avoid fragmentation or to at least reduce the number of fragments, which adds overhead to LoRaWAN packets.

Table 8 summarizes the analysis results comparing different schemes used for data transmission provided in Section 4. The first two rows of the table compare the schemes used for the headers in the approaches. This way, comparison between CoAP/UDP/IP with HTTP/TCP/IP header sizes for different transactions of Swarm over LoRaWAN show reduction of at least 78.61%, and up to 88.83%. Similarly, comparison between SCHC and HTTP/TCP/IP header sizes for different transactions show reduction of at least 97.51% and up to 98.70%. Therefore, there is a significant header size reduction in SCHC compared to the HTTP/TCP/IP. Moreover, the table also compares CBOR with JSON payload size, which shows that CBOR reduces the payload size up to 50% in our example.

Additionally, Table 9 summarizes the analysis results comparing different approaches provided in Section 4 with HTTP/TCP/IP header and JSON payload. As expected, we can see that the approach using SCHC header compression and CBOR data format can reduce the packet sizes even more than the approach using CoAP and CBOR schemes. This approach of using SCHC and CBOR has significant packet size reduction of 98.18% compared to the approach of using HTTP and JSON serialization and this reduction for the approach which uses CoAP and CBOR schemes is 88.57%. It is important to highlight that, while securing the packets with COSE costs 115 bytes (for ECDH-SS-EdDSA in our approaches), there is a big difference between packet sizes of secured approaches with unsecured approach of HTTP and JSON. The approach using SCHC and CBOR secured with COSE can decrease by up to 68.31% and the approach using CoAP and CBOR secured with COSE can decrease by up to 58.70% compared with the approach using HTTP and JSON. the reason that we used COSE. In a future work, we plan to analyze OSCORE instead. The reason that we used COSE in this analysis work is that OSCORE only supports symmetric cryptography while in the Swarm we use asymmetric cryptography. If we were considering OSCORE, we would have a smaller security overhead; however, we would have no support for algorithms such as ECDH-SS, which are needed in the Swarm. Therefore, in our next steps, we could analyze OSCORE instead, especially when considering more generic constructs (i.e., not necessarily using the Swarm). We also highlight this point that the typical header size of IPv6 packet is 40 bytes, which means 20 bytes more than that for IPv4. This difference could constantly affect all the SCHC analysis, having more than 20 bytes or causing one more fragmentation.

Table 10 compares the number of LoRaWAN fragments in different approaches through our testing scenario. In this scenario, downlink LoRaWAN fragments are the Discovery Request (3-b), Negotiation Request (5-b), Agreement Request (7-b), Confirm Response (7-d) and Use Request (9-b), and uplink ones are the Discovery Response (3-b), Negotiation Response (5-b), Agreement Response (7-b), Confirm Request (7-d) and Use Response (9-b). One can see in Table 10 that the approach of using CoAP/UDP/IP and CBOR schemes and the one using SCHC and CBOR has the same fragmentation number of packets. Considering results for Table 9 and Table 10, while the numbers of LoRaWAN fragments are the same for the approach of using CoAP/UDP/IP and CBOR schemes and the one using SCHC and CBOR but, in the scenario of SCHC and CBOR, the length of each packet is smaller which leads to decrease the delay and interference in the network and causes the supporting of more services in the constrained network.

The results of Table 10 also show that the approach of using CoAP/UDP/IP and CBOR schemes and the one using SCHC and CBOR have the least fragmentation number of packets, which are eight downlink and nine uplink packets. This is a significant improvement but not enough for LoRaWAN plans currently available. For example, in a commercial plan in Brazil, currently, there is a limitation of eight number of downlink packets and 160 number of uplink packets transmission per day. Therefore, the achieved result for the number of downlink packets, shown in Table 10, is not yet enough for this kind of plan that is available now. Therefore, as a future work, we plan to investigate, propose and analyze different network architectures and assess the suitability of the proposal in constrained devices. 

## Figures and Tables

**Table 1:** State of the art comparison.

	Thielemans et al. [18]	Weber et al. [19]	Sanchez et al. [20]	Lara et al. [21]	Abdelfadeel et al. [22]	Sanchez et al. [23]
**Application layer**	CoAP	CoAP	CoAP	CoAP	CoAP	CoAP
**Transport layer**	UDP	UDP	UDP	UDP	UDP	UDP
**Network layer**	IP	IP	IP	IP	IP	IP
**Adaptation layer**	6LoWPAN	6LoRaWAN	SCHC	SCHC	SCHC	SCHC
**Serialization** **format**	-	-	-	CBOR	CBOR	CBOR
**Security**	-	-	-	-	COSE	EDHOC
**Analysis/** **Performance** **evaluation**	Range measurement	Feasibility of interoperability	- Transmission time - Packet delivery rate - End device processor, - Memory overhead - Overall power consumption	Transmission time	- Transmission time - Reliability	- Exploring the security flaws - Feasibility of interoperability

**Table 2:** Communication load in terms of packet sizes in byte for transactions where it affects on the LoRaWAN network considering the HTTP/TCP/IP and JSON scheme.

Packet Parts	Discovery (3-b)		Negotiation (5-b)		Agreement (7-b)		Confirm (7-d)		Use (9-b)	
	Req.	Res.	Req.	Res.	Req.	Res.	Req.	Res.	Req.	Res.
**HTTP Header**	231	333	200	200	152	331	166	155	209	149
**TCP Headr**	32	32	32	32	32	32	32	32	32	32
**IP Header**	20	20	20	20	20	20	20	20	20	20
**Total Header**	**283**	**385**	**252**	**252**	204	**383**	218	207	**261**	201
**JSON Payload**	191	**746**	**958**	**958**	119	2	**0**	**1036**	**0**	20
**Total Packet**	**474**	**1131**	**1210**	**1210**	**323**	**385**	218	**1242**	**261**	221
**Number of LoRaWAN Fragments**	**2**	**5**	**5**	**5**	**2**	**2**	**1**	**6**	**2**	**1**

**Table 3:** Communication load in terms of packet sizes in bytes for transactions where it affects on LoRaWAN network considering CoAP/UDP/IP and JSON schemes.

Packet Parts	Discovery (3-b)		Negotiation (5-b)		Agreement (7-b)		Confirm (7-d)		Use (9-b)	
	Req.	Res.	Req.	Res.	Req.	Res.	Req.	Res.	Req.	Res.
**Total Header** **(CoAP 15, UDP 8 and IP 20 bytes)**	43	43	43	43	43	43	43	43	43	43
**JSON Payload**	191	**746**	**958**	**958**	119	2	0	**1036**	0	20
**Total Packet**	234	**789**	**1001**	**1001**	162	45	43	**1079**	43	63
**Number of LoRaWAN Fragments**	**1**	**4**	**5**	**5**	**1**	**1**	**1**	**5**	**1**	**1**

**Table 4:** Communication load in terms of packet sizes in bytes for transactions where it affects on LoRaWAN network considering CoAP/UDP/IP and CBOR schemes.

Packet Parts	Discovery (3-b)		Negotiation (5-b)		Agreement (7-b)		Confirm (7-d)		Use (9-b)	
	Req.	Res.	Req.	Res.	Req.	Res.	Req.	Res.	Req.	Res.
**Total Header** **(CoAP 15, UDP 8 and IP 20 bytes)**	43	43	43	43	43	43	43	43	43	43
**CBOR Payload**	157	**635**	**843**	**843**	113	1	0	**916**	0	15
**Total Packet**	200	**678**	**886**	**886**	156	44	43	**959**	43	43
**Number of LoRaWAN Fragments**	**1**	**3**	**4**	**4**	**1**	**1**	**1**	**4**	**1**	**1**

**Table 5:** Communication load in terms of packet sizes in byte for transactions where it affects on the LoRaWAN network considering SCHC and CBOR schemes.

Packet Parts	Discovery (3-b)		Negotiation (5-b)		Agreement (7-b)		Confirm (7-d)		Use (9-b)	
	Req.	Res.	Req.	Res.	Req.	Res.	Req.	Res.	Req.	Res.
**SCHC Header**	5	6	6	6	5	5	5	6	5	5
**CBOR Payload**	157	**635**	**843**	**843**	113	1	0	**916**	0	15
**Total Packet**	162	**641**	**849**	**849**	118	6	5	**922**	5	20
**Number of LoRaWAN Fragments**	**1**	**3**	**4**	**4**	**1**	**1**	**1**	**4**	**1**	**1**

**Table 6:** Communication load in terms of packet sizes in bytes for transactions where it affects on the LoRaWAN network considering COSE, CoAP/UDP/IP and CBOR schemes.

Packet Parts	Discovery (3-b)		Negotiation (5-b)		Agreement (7-b)		Confirm (7-d)		Use (9-b)	
	Req.	Res.	Req.	Res.	Req.	Res.	Req.	Res.	Req.	Res.
**Total Header** **(COSE 115, CoAP 15, UDP 8 and IP 20 bytes)**	158	158	158	158	158	158	158	158	158	158
**CBOR Payload**	157	635	843	843	113	1	0	916	0	15
**Total Packet**	315	**793**	**1001**	**1001**	271	159	158	**1074**	158	173
**Number of LoRaWAN Fragments**	**2**	**4**	**5**	**5**	**2**	**1**	**1**	**5**	**1**	**1**

**Table 7:** Communication load in terms of packet sizes in bytes for transactions where it affects on the LoRaWAN network considering COSE, SCHC and CBOR schemes.

Packet Parts	Discovery (3-b)		Negotiation (5-b)		Agreement (7-b)		Confirm (7-d)		Use (9-b)	
	Req.	Res.	Req.	Res.	Req.	Res.	Req.	Res.	Req.	Res.
**Total Header** **(COSE 115 and SCHC 6 bytes)**	121	121	121	121	121	121	121	121	121	121
**CBOR Payload**	157	635	843	843	113	1	0	916	0	15
**Total Packet**	278	**756**	**964**	**964**	234	122	121	**1037**	121	136
**Number of LoRaWAN Fragments**	**2**	**4**	**4**	**4**	**1**	**1**	**1**	**5**	**1**	**1**

**Table 8:** Comparison results among different schemes for data transmission.

Scheme to Compare with	Applied Scheme	Discovery (3-b) [%]		Negotiation (5-b) [%]		Agreement (7-b) [%]		Confirm (7-d)		Use (9-b) [%]	
		Req.	Res.	Req.	Res.	Req.	Res.	Req.	Res.	Req.	Res.
**HTTP/TCP/IP Header**	**CoAP/UDP/IP Header**	84.81	**88.83**	82.94	82.94	78.92	88.77	80.28	79.23	83.52	78.61
	**SCHC Header**	98.23	**98.70**	98.02	98.02	97.55	98.69	97.71	97.58	98.08	97.51
**JSON Payload**	**CBOR Payload**	17.80	14.88	12.00	12.00	5.04	**50.00**	0	11.58	0	25

**Table 9:** Comparison results between proposed approaches with the approach using HTTP and JSON serialization for data transmission.

Approaches	Discovery (3-b) [%]		Negotiation (5-b) [%]		Agreement (7-b) [%]		Confirm (7-d) [%]		Use (9-b) [%]	
	Req.	Res.	Req.	Res.	Req.	Res.	Req.	Res.	Req.	Res.
**SCHC and CBOR**	65.61%	43.32%	29.83%	29.83%	63.16%	**98.18%**	97.25%	**25.82%**	97.70%	90.50%
**SCHC and CBOR** **with COSE Security**	41.35%	33.16%	20.33%	20.33%	27.55%	**68.31%**	44.50%	**16.57%**	53.64%	38.46%
**CoAP/UDP/IP and CBOR**	57.81%	40.05%	26.78%	26.78%	51.70%	**88.57%**	80.28%	**22.85%**	83.52%	73.76%
**CoAP/UDP/IP and CBOR** **with COSE security**	33.54%	29.89%	17.27%	17.27%	16.10%	**58.70%**	27.52%	**13.60%**	39.46%	21.72%

**Table 10:** Number of fragments transmitted through the LoRaWAN network.

Approaches	Downlink	Uplink
**HTTP/TCP/IP and JSON**	17	14
**CoAP/UDP/IP and JSON**	13	12
**CoAP/UDP/IP and CBOR**	**11**	**9**
**SCHC and CBOR**	**11**	**9**
**CoAP/UDP/IP and CBOR** **with COSE Security**	15	11
**SCHC and CBOR** **with COSE Security**	13	10
